# The effect of Kuiyuan chewing tablet on hyperuricemia: protocol for a randomized, double-blind, multicenter, parallel-controlled trial

**DOI:** 10.3389/fendo.2025.1517009

**Published:** 2025-04-30

**Authors:** Man Han, Chuanhui Yao, Yuting Huang, Jianyong Zhang, Jing Yu, Xinliang Lu, Yu Xue, Xiaopo Tang, Hejian Zou, Quan Jiang

**Affiliations:** ^1^ Department of Rheumatology, Guang’anmen Hospital, China Academy of Chinese Medical Sciences, Beijing, China; ^2^ School of Chinese Medicine, Beijing University of Chinese Medicine, Beijing, China; ^3^ Department of Rheumatology, Shenzhen Traditional Chinese Medicine Hospital, Shenzhen, China; ^4^ Department of Rheumatology, Affiliated Hospital of Liaoning University of Traditional Chinese Medicine, Shenyang, China; ^5^ Department of Rheumatology, Inner Mongolia Autonomous Region Traditional Chinese Medicine Hospital, Hohhot, China; ^6^ Department of Rheumatology and Immunology, Huashan Hospital, Fudan University, Shanghai, China

**Keywords:** hyperuricemia, Kuiyuan chewing tablet, uric acid lowering therapy, multicenter randomized controlled trial, protocol

## Abstract

**Background:**

Hyperuricemia (HUA) is a metabolic disorder characterized by elevated serum uric acid levels resulting from dysfunction in purine metabolism and/or inadequate uric acid excretion. It is an independent risk factor for many chronic diseases and is associated with a poor long-term prognosis. Existing uric acid-lowering drugs often lead to numerous adverse reactions, resulting in poor patient compliance and limited clinical application. Therefore, this study aims to investigate the effect of Kuiyuan Chewing Tablet (KYCT) on serum uric acid (SUA) levels in patients with HUA, and to seek a safe alternative therapy for reducing uric acid.

**Methods and analysis:**

This study is a multicenter, randomized, double-blind, parallel-controlled trial. HUA patients who meet the inclusion criteria will be randomly assigned in a 1:1 ratio to either (1) the control group (placebo of KYCT, specifications: 0.3g per tablet, 1.2g per dose, twice a day, taken with warm water 30 minutes after meals) or (2) the experimental group (KYCT, specifications: 0.3g per tablet, 1.2g per dose, twice a day, taken with warm water 30 minutes after meals). Both groups will receive dietary control, comorbidity prevention, and health education during the intervention period. The primary outcome will be the proportion of subjects with SUA levels <420 umol/L. Secondary outcomes will include the proportion of subjects with SUA levels <360 umol/L, the percentage change in SUA levels from baseline to each visit, the maximum percentage change in SUA levels from baseline to the third month, the number of gout attacks, changes in body measurements (weight, waist circumference, hip circumference, BMI), blood pressure, blood lipids, fasting blood glucose levels, and the proportion of subjects reporting gout attacks (cumulative up to each visit). Each group of patients will be assessed at baseline, as well as at the 4th, 8th, and 12th weeks.

**Discussion:**

This study aims to evaluate the effects of a 12-week treatment with KYCT on patients with HUA. We hypothesize that compared to placebo, KYCT would significantly improve SUA levels without provoking significant adverse reactions. These findings potentially pave the way for a safe and effective alternative therapy for HUA.

## Background

Hyperuricemia (HUA) is a metabolic disorder resulting from disturbances in purine metabolism and/or impaired uric acid excretion. It is clinically diagnosed when the serum uric acid level exceeds 420 µmol/L (360 µmol/L for females) on two separate occasions (1). Relevant data suggest that the incidence of HUA and gout has been increasing year by year, with a trend toward younger age groups, due to changes in lifestyle and dietary patterns. HUA is now the second most common metabolic disorder after diabetes (2). Reports indicate that the prevalence of HUA varies among ethnic groups, ranging from 2.6% to 36% (3–5). Large-scale epidemiological investigations in mainland China have revealed that the prevalence of HUA in the general population is 17.4%, ranging from 15.5% to 24.6% in different regions and exhibiting an increasing trend (6). However, the reported prevalence is significantly higher in some countries. For instance, the prevalence of HUA is 21.2% for men and 21.6% for women in the United States (7) while it is 25.8% (34.5% for men and 11.6% for women) in Japan (8).

HUA is a global illness that can lead to gout, with some severe cases resulting in tophi, joint deformities, and complications such as kidney and cardiovascular disease. Numerous studies suggest that HUA is an independent risk factor for chronic kidney disease, hypertension, cardiovascular disease, and diabetes, and that it is linked to poor long-term outcomes (9–13). Elevated blood uric acid levels are the primary cause of HUA and associated complications, underscoring the vital necessity for effective uric acid level control. Pharmacological treatment is recommended for asymptomatic HUA patients in Asian countries such as Japan and China (14). According to Chinese guidelines (2), urate-lowering therapy should be initiated in asymptomatic HUA patients with serum uric acid levels of ≥ 540 μmol/L or ≥ 480 μmol/L with comorbid conditions like hypertension, dyslipidemia, and diabetes.

According to Chinese guidelines, Allopurinol, Febuxostat, and Benzbromarone are the first-line drugs for lowering uric acid levels (2). Allopurinol is particularly suitable for patients with increased uric acid production, but it might cause fatal allergic reactions in Asian populations (15, 16). Allopurinol is metabolized by the kidneys and must be started at a low dose when renal function is normal, limiting its clinical application (17). Studies have shown that Febuxostat, another uric acid-lowering drug, may increase the risk of cardiovascular disease and may also induce gout attacks (18). Benzbromarone is especially useful for patients with HUA and gout who have decreased uric acid excretion, but caution should be exercised in patients with renal insufficiency and kidney stones. Moreover, it should be supplemented with a large amount of water intake to increase excretion and alkalize the urine. Caucasians have reportedly experienced acute liver failure as a result (19). Low compliance among patients taking uric acid-lowering medications is a result of the safety issues with these medications and the underdiagnosis of the disease among patients. The results of a 2018 meta-analysis showed that the average compliance of uric acid-lowering drugs was 47% (ranging from 17% to 83.5%) (20).

Therefore, it is crucial to develop new medication for HUA that can lower blood uric acid levels efficiently with minimal side effects. Exploring natural therapeutic agents from medicinal plants that have high efficacy and low toxicity is a promising alternative treatment option. In recent years, edible Sunflower (Helianthus annuus L.), a member of the Asteraceae family and a native of North America, has been widely cultivated in China, Russia, Argentina, France, and other countries (21). Kuiyuan Chewing Tablet(KYCT) is extracted from the heads of the sunflower plant, which mainly contains flavonoids, coumarins, and phenolic acids. It helps lower uric acid levels, reduces inflammation and pain, repairs the liver and kidneys, and is widely used as a dietary health product in China. Preclinical studies indicate that KYCT’s primary active component, sunflower head extract (SHE), exerts dual-pathway hypouricemic effects. First, SHE suppresses uric acid synthesis by inhibiting xanthine oxidase (XO) activity via its polyphenolic constituents, as evidenced by 32-41% XO activity reduction in hyperuricemic models (22). Second, SHE enhances uric acid excretion through coordinated regulation of urate transporters: downregulating reabsorption transporters (URAT1/GLUT9) and upregulating secretory transporter ABCG2, thereby shifting renal/intestinal transport toward net excretion (23). These synergistic mechanisms may explain KYCT’s preclinical efficacy comparable to allopurinol and benzbromarone, though clinical validation remains warranted.

In this study, we aim to investigate the effect of Sunflower Bud Chewing Tablet on serum uric acid (SUA) in patients with HUA and to seek safe alternative therapy for uric acid reduction. We will conduct a randomized parallel-controlled trial to determine the impact of KYCT on patients with HUA. We hypothesize that KYCT would significantly improve SUA levels without provoking significant adverse reactions compared to placebo. In addition, these findings potentially pave the way for a safe and effective alternative therapy for HUA.

## Materials and methods

### Study objective

This multicenter, randomized, double-blind, placebo-controlled trial aims to investigate the efficacy and safety of KYCT for reducing serum uric acid (SUA) levels in patients with HUA. We will conduct a 12-week, multicenter, double-blind, randomized controlled trial to ascertain its effectiveness in treating HUA. We hypothesize that KYCT will significantly outperform a placebo to lower SUA levels in patients with HUA over 12 weeks of treatment.

### Study design

**Figure 1:**
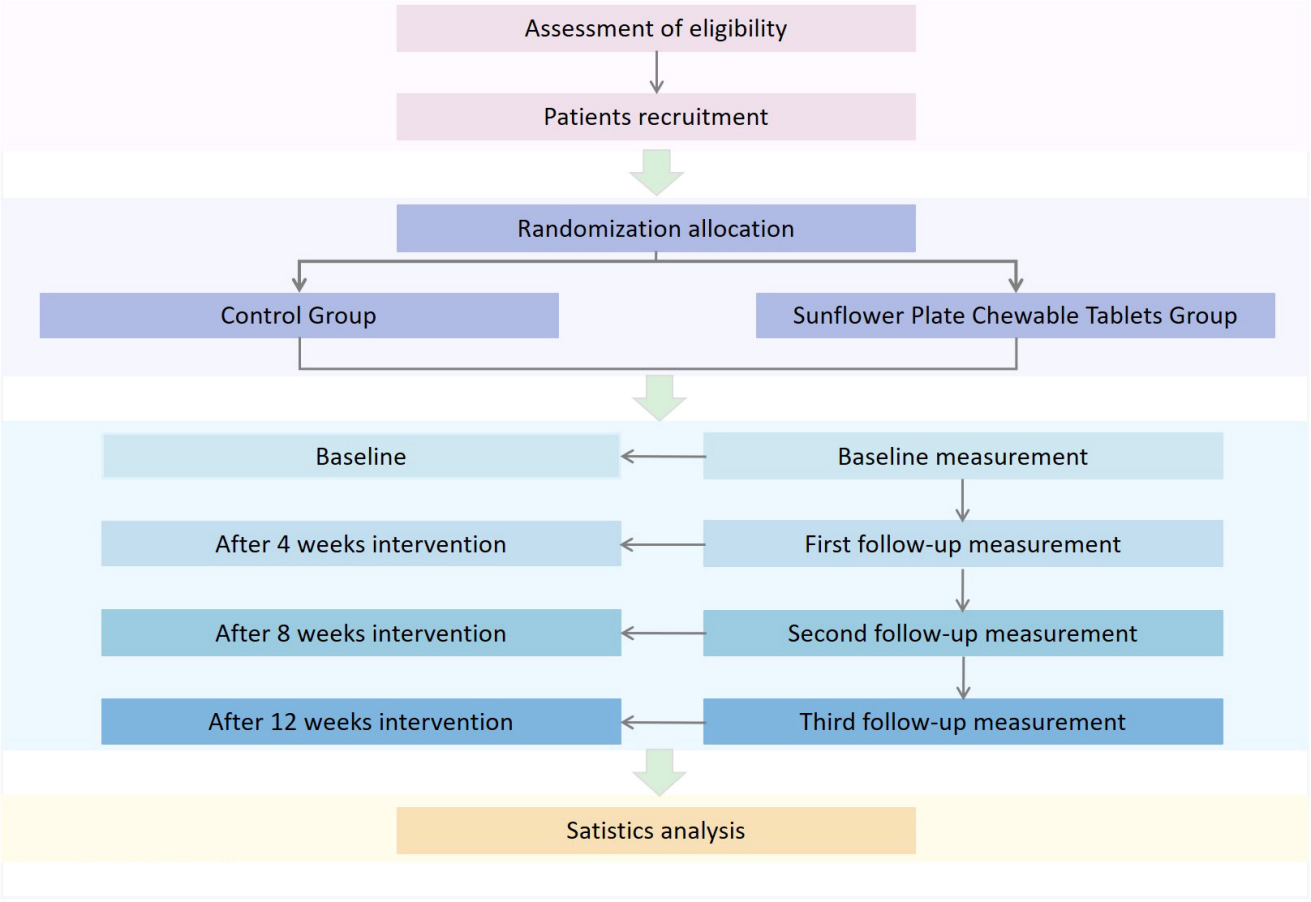
Trial flow chart.

This randomized, double-blind, multicenter, parallel-controlled trial started and ended recruitment in July 2023. The 1-year study will randomize participants into experimental and control groups. Assessments will be conducted at baseline, weeks 4, 8, and 12. The study flowchart is shown in **Figure 1**. This study has been approved by the Ethics Review Committee of Guang’anmen Hospital, China Academy of Chinese Medical Sciences (approval No.2023-057-YW). Written informed consent will be obtained from all patients prior to enrollment. This study will follow the Consolidated Standards of Reporting Trials (CONSORT) guidelines (24).

### Study setting

This study will recruit 126 eligible participants from multiple medical institutions, including Guang’anmen Hospital of China Academy of Chinese Medical Sciences, Huashan Hospital affiliated to Fudan University, Shenzhen Traditional Chinese Medicine Hospital, Inner Mongolia Autonomous Region Hospital of Traditional Chinese Medicine, and Liaoning University of Traditional Chinese Medicine Affiliated Hospital.

### Inclusion criteria

(1) Age 18–70 years (at the time of obtaining informed consent), any gender; (2) Meet the diagnostic criteria for asymptomatic HUA; (3) Blood uric acid level > 8.0 mg/dL (480 μmol/L) at enrollment after 1 month or longer of low purine dietary control; (4) No history of acute gout attack before enrollment; (5) Normal electrocardiogram at enrollment; (6) Able to swallow Tablet; (7) Voluntarily participate, able to comply with regular follow-up and complete patient logs, and sign an informed consent form.

### Exclusion criteria

(1) Secondary HUA caused by renal disease, hematological disorders, medication, tumor radiotherapy/chemotherapy, organ transplantation, or other reasons; (2) Use of allopurinol, febuxostat, benzbromarone, probenecid, or Chinese herbal medicine with uric acid-lowering effect within 4 weeks prior to enrollment; (3) History of stroke, transient ischemic attack, myocardial infarction, heart failure (NYHA class II-IV), or coronary artery surgery (such as angioplasty, stenting, or bypass surgery); (4) Abnormal liver function tests with aspartate aminotransferase (AST), alanine aminotransferase (ALT), or γ-glutamyltransferase (GGT) levels higher than 1.5 times the upper limit of normal at screening (Visit 1); (5) Abnormal renal function tests with serum creatinine (SCr) levels higher than 1.5 times the upper limit of normal at screening (Visit 1); (6) Co-existing malignant tumors or psychiatric disorders; (7) Known allergy or intolerance to any component of Kuiyuan chewing tablet or the investigational medicinal product excipients; (8) Known hypersensitivity or history of severe asthma exacerbated by nonsteroidal anti-inflammatory drugs (NSAIDs) or aspirin, or any component of NSAIDs or aspirin; (9) Use of oral corticosteroids for 10 or more consecutive days or use of intramuscular, subcutaneous, or intra-articular corticosteroids within 1 month prior to screening (Visit 1); (10) Co-existing autoimmune diseases; (11) Use or need for anticoagulants (such as aspirin, other salicylates, heparin, or warfarin) or antiplatelet agents (such as clopidogrel or ticlopidine); (12) Use or need for diuretics; (13) History of alcohol or drug abuse or daily use of analgesics for any reason; (14) Pregnancy, lactation, or planned pregnancy within 3 months after the last dose of investigational medicinal product; (15) Participation in other clinical studies within 3 months prior to screening (Visit 1); (16) Other conditions deemed unsuitable for this clinical study by the investigator.

### Withdrawal criteria

#### Decision made by the investigator

1) Occurrence of allergic reactions or serious adverse events that require discontinuation of the trial according to the investigator’s judgment; 2) Occurrence of other complications or physiological changes during the trial that make it inappropriate for the participant to continue; 3) Poor compliance by the participant; 4) other situations deemed inappropriate for the participant to continue by the investigator or in which continuing the trial is difficult according to the investigator’s judgment.

#### Participant self-withdrawal

1) Participants who are unwilling or unable to continue with the clinical trial for any reason and request to withdraw from the trial are considered to have self-withdrawn;
2) Participants who do not explicitly request to withdraw but do not receive treatment or participate in follow-up visits are considered lost to follow-up.

### Handling of dropout cases

Subjects who withdraw prematurely from the trial or are lost to follow-up will be contacted to complete a final assessment. Every effort will be made to collect efficacy and safety data for these subjects. Reasons for premature withdrawal will be documented in the case report form and trial conclusion form.

### Ineligibility criteria

(1) Incorrectly enrolled subjects who did not meet all inclusion criteria or met any exclusion criteria; (2) Subjects who met inclusion criteria but did not take the study medication or have no follow-up data; (3) Subjects who switched medications or added unspecified co-medications, especially those that could significantly impact assessment of efficacy and safety.

### Termination criteria

(1) Serious safety issues occurred during the trial; (2) Lack of clinically significant efficacy demonstrated for the study medicine during the trial; (3) Major errors in trial design or significant deviations from protocol that preclude efficacy evaluation; (4) Sponsor request for termination due to funding, management issues, or other reasons; (5) The China Food and Drug Administration orders termination of the trial.

### Random allocation and blinding

A double-blind, single-mimic approach was chosen because the placebo Tablet in the control group had the same appearance, specifications, size, and odor as the Kuiyuan chewable Tablet. All test drugs were provided by Beijing Qino Biotechnology Co., Ltd. and had uniform internal and external packaging.

A two-level blind design was adopted, with the first level corresponding to the group (such as Group A and Group B) for each case number and the second level corresponding to the treatment (test group and control group) for each group. The Institute of Basic Clinical Medicine of Traditional Chinese Medicine, China Academy of Chinese Medical Sciences, established the random coding table. The two-level blind bottoms were separately sealed, with two copies each, and stored at the Guang’anmen Hospital of the China Academy of Chinese Medical Sciences and Beijing Qino Biotechnology Co., Ltd., respectively.

Each coded investigational drug has a corresponding emergency letter, which contains a piece of paper indicating the type of drug that code is to be used in case of emergency unblinding. The emergency letter should be sealed and sent along with the investigational drug of the corresponding code to each clinical research center, where it should be kept by the center and not opened unless necessary. If a severe adverse event occurs or a subject needs to be rescued and it is necessary to know the treatment the subject received, the investigator may unblind the subject. Once unblinded, the subject will be withdrawn from the trial and treated as a dropout, and the reason for withdrawal should be recorded in the case report form by the investigator. All emergency letters will be collected along with the case report form at the end of the trial for the unblinding audit.

A two-step unblinding method was used. The data were locked after all case report forms were entered into the database, and the data underwent questioning, verification, and blinding audits. The investigator who kept the blind code then performed the first unblinding, revealing groups A and B. A biostatistician entered The data into the computer and linked it to the data file for statistical analysis. After the statistical analysis was completed, a second unblinding was performed to identify the treatment groups and the experimental and control groups.

### Sample size calculation

According to the literature of Dalbeth N (25), Zhang X (26) et al., assuming that the effective rate of the experimental group is about 60% and the effective rate of the control group is about 30%, the sample size with a power of 0.8 was calculated to detect the difference between the two groups in terms of the effective rate, using the formula for comparing two sample proportions (27). The power of the test (1-β) was set at 0.8, and the significance level (Type I error rate) was set at 0.05 for a two-sided test. The sample size was estimated to be 53 per group, considering a dropout rate of 15%, resulting in a total of 126 participants, with 63 participants per group.

### Recruitment

#### Training of participants

Prior to the start of the clinical trial, the supervisor and the person in charge of each trial center should conduct training for the participants on the trial protocol in order to make the participants understand and be familiar with the nature, effects, efficacy, and safety of the trial drug (including relevant information on the preclinical studies of the drug), and also to grasp all the new information related to the drug that is found during the conduct of the clinical trial.

### Quality control

Both sponsors and participants should adopt standard operating procedures to ensure quality control and the implementation of the quality assurance system of the clinical trial. All observations and findings in clinical trials should be verified to ensure the data’s reliability and that all conclusions in clinical trials are derived from the original data. Quality control must be applied at every data processing stage to ensure that all data are reliable and correctly processed.

### Monitoring

The sponsor appoints a monitor to conduct regular on-site monitoring visits to the trial hospital to ensure that all elements of the study protocol are strictly adhered to and that the original data are checked to ensure consistency with the CRF form.

### Data management

The participant will complete the case report form and then checked and approved by the clinical supervisor before being delivered to the full-time data manager to set up a database. The data on the CRF form will be entered into the database independently in duplicate by two data entry personnel, reviewed manually, checked by the computer, and then blinded before the data are locked for statistical analysis.

### Intervention measures

#### Control group

The control group received placebo Tablet that were identical in appearance to the KYCT. Placebo Tablet, which weigh 0.3g per tablet, were manufactured by Beijing Qinuo Biotechnology Company Limited. Participants took 1.2g placebo (4 Tablet) twice daily with warm water 30 minutes after meals.

#### Intervention group

The intervention group received KYCT weighing 0.3g per tablet, manufactured by Beijing Qinuo Biotechnology Company Limited. Participants took 1.2g per dose (4 Tablet) twice daily, with warm water 30 minutes after meals.

During the trial, all participants received standard care, including (1) dietary control, including adopting a low-calorie diet, maintaining ideal body weight, avoiding high-purine foods, strictly abstaining from all kinds of alcohol, and drinking at least 2000 ml of water per day; (2) trigger avoidance, including quitting smoking, avoiding overeating, avoiding excessive fatigue and mental stress, and avoiding the use of drugs that severely affect uric acid excretion, such as some diuretics; (3) prevention and treatment of comorbidities, including hyperlipidemia, diabetes, hypertension, coronary heart disease, cerebrovascular disease, etc.

During the trial, all participants were provided with a uniform treatment medication for gout attacks: 60 mg of Etoricoxib Tablet (manufactured by Hangzhou MSD Pharmaceutical Co., Ltd., China, 60 mg/tablet, Batch No. J20180059), taken once daily, with a 7-day supply provided to patients at the time of enrollment.

The study medication was immediately discontinued if SUA levels were <180 umol/L during the visit.

### Result assessment

Evaluators assessed all relevant indicators at baseline, 4th, 8th, and 12th week. Details of the assessments are provided in **Table 1**.

**Table 1:** Schedule for data collection, the process of the assessments per visit.

measures	Baseline	Intervention phase		
Time(weeks)	-2-0	4	8	12
Informed consent	**✓**			
Demographics and Past medical history	**✓**			
Physical examination	**✓**	**✓**	**✓**	**✓**
Laboratory Tests	**✓**	**✓**	**✓**	**✓**
Electrocardiogram	**✓**			**✓**
Acertainment of Inclusion and Exclusion Table	✓			
Distribution of the study medicine		✓	✓	
Drug recovery		✓	✓	✓
Routine follow-up measurement table		✓	✓	✓
Adverse events		✓	✓	✓
Endpoint events		✓	✓	✓
Medication adherence assessment		✓	✓	✓

#### Screening criteria

Urine pregnancy tests were performed in premenopausal female subjects and were evaluated before medication and at week 12 of medication.

#### Efficacy endpoints

##### Primary efficacy endpoint

The proportion of participants who maintained SUA levels <420 umol/L at week 12 (SUA levels were uniformly determined by uricase-peroxidase coupling assay, as described in Appendix 1).

##### Secondary efficacy endpoints

Proportions of participants maintaining sUA level <360 umol/L at week 12; percent change of sUA level from baseline to each visit; maximal percent change of sUA level from baseline to week 12; changes in anthropometric parameters (body weight, waist circumference, hip circumference, BMI), blood pressure, lipid profile, and fasting blood glucose level; number of gout flares reported; proportion of participants with gout flares cumulatively assessed at each visit.

##### Prespecified exploratory endpoint

The percentage of participants achieving a minimal clinically important difference (MCID) in serum uric acid (sUA) reduction (≥1.5 mg/dL from baseline) is noteworthy. This threshold is based on the following considerations:

1. Gout flare prevention: A reduction of ≥1.0 mg/dL is associated with a 50% decrease in flare risk (28).
2. Renal protection: A reduction of ≥1.5 mg/dL is correlated with a diminished decline in estimated glomerular filtration rate (eGFR) in individuals with chronic kidney disease (29).

#### Safety endpoints

Vital signs (body temperature, respiratory rate, heart rate, blood pressure), liver function, renal function, complete blood count, and urinalysis indicators will be evaluated at baseline, weeks 4, 8, and 12 during the study period. A 12-lead electrocardiogram will be evaluated at baseline and week 12 during the study period.

All blood samples were collected after a 12-hour fast (refrain from eating or drinking) between 8:00 and 10:00 a.m. Blood samples will be collected at each participating center; all centers are tertiary hospitals with reliable laboratory facilities. All samples were processed promptly at the local tertiary hospital laboratories.

### Data analysis

The complete analysis set (FAS) and per protocol set (PPS) were used for efficacy evaluation analysis. The safety set (SS) was used for safety indicator analysis; since concomitant medications may affect efficacy and safety, the overall combination therapy was analyzed using the SS. General data was analyzed using the FAS and PPS. The last observation was carried forward for efficacy analysis for participants who withdrew prematurely. Dropouts with baseline data were included in efficacy analyses using the last observation carried forward. Excluded cases were not included in the efficacy statistical analysis, but those who received at least one treatment and had at least one safety record participated in the adverse reaction analysis.

To address potential confounding from lifestyle modifications, we will implement a three-pronged strategy to differentiate the specific effects of KYCT from those attributable to dietary control and health education. First, the double-blind placebo-controlled design ensures both groups receive identical lifestyle interventions, with KYCT administration being the sole therapeutic distinction between arms. Second, our primary analysis will employ analysis of covariance (ANCOVA) adjusting for key lifestyle covariates including physical activity levels (measured in MET-minutes/week) and daily purine intake (quantified through 24-hour dietary recalls). Third, sensitivity analyses will examine treatment effects in predefined adherent subgroups (≥80% protocol compliance for both medication intake and lifestyle recommendations) to assess outcome robustness against potential lifestyle adherence variations.

Furthermore, to address potential heterogeneity in treatment effects across subpopulations, three methodological safeguards have been incorporated: First, stratified randomization by baseline sUA tertiles (7.0-7.5, 7.6-8.5, >8.5 mg/dL) and sex ensures balanced subgroup distributions. Second, pre-specified Bayesian hierarchical models with regularizing priors [N(0,1)] will estimate subgroup effects while pooling information across multicenter clusters. Third, all subgroup comparisons will apply Benjamini-Hochberg FDR correction (<10%) within stratification categories, with results explicitly interpreted as exploratory hypotheses per CONSORT extension guidelines.

Statistical analysis was performed using SPSS 20.0 software. We presented the results as mean ± standard deviation for quantitative data that followed a normal distribution. We used an independent sample t-test to compare differences between the two groups and a paired sample t-test to compare differences within each group before and after the intervention. We presented the results as the median and interquartile range for quantitative data that followed a skewed distribution. We used the Mann-Whitney test to compare differences between the two groups and the Wilcoxon test to compare differences within each group before and after the intervention. We used Spearman correlation analysis to examine the correlation between variables. A significance level of P < 0.05 was considered statistically significant.

The proportion of participants attaining the MCID (≥1.2 mg/dL sUA reduction) will be compared between treatment arms using logistic regression, adjusted for baseline sUA levels and stratification factors (sex, baseline sUA tertiles). Results will be interpreted as hypothesis-generating, with 95% confidence intervals reported to quantify precision. Sensitivity analyses will assess MCID consistency across predefined subgroups (baseline sUA strata, renal function categories).

## Discussion

Different countries have varying guidelines for managing asymptomatic hyperuricemia. This condition is challenging to treat due to limited indications and the potential adverse effects of first-line uric acid-lowering drugs like febuxostat and allopurinol. Consequently, there exists a pressing need for the development of alternative therapies that are both safer and more efficacious. Historical texts from ancient Chinese medicine highlight the therapeutic potential of the sunflower head (Helianthus annuus), attributing to it properties that enable the clearing of heat and alleviation of pain. This botanical has been traditionally employed in the treatment of various ailments, including hypertension, dysmenorrhea, and headaches. Recent scientific inquiries have substantiated the uric acid-lowering and anti-gout capabilities of the sunflower head, identifying flavonoids, coumarins (notably, scopolamine), and phenolic acids as the principal bioactive components responsible for its efficacy. These compounds have been shown to exert a dual action, simultaneously mitigating uric acid levels and offering anti-inflammatory and analgesic benefits. Building upon these findings, a novel pharmaceutical agent, referred to as KYCT, has been formulated, incorporating flavonoids, coumarins (including scopolamine), and phenolic acids as its core active ingredients. This drug has successfully progressed through Phase II clinical trials in China and has been included in the research project of the National Health Commission.

This study aims to clarify the therapeutic efficacy of KYCT on asymptomatic hyperuricemia and provide more evidence-based treatment options for clinical practice. Recent investigations have explored the potential therapeutic effects of sunflower seed head extract on hyperuricemia (HUA). A seminal study by Lanzhou Li and colleagues (22) demonstrated that administration of sunflower seed head extract to HUA-afflicted mice resulted in a significant reduction in uric acid (UA) levels, alleviation of ankle swelling, and mitigation of urate crystal deposition. Further research has elaborated on these findings, indicating that sunflower head enzyme hydrolysate may attenuate uric acid concentrations through the inhibition of xanthine oxidase (XOD), adenosine deaminase (ADA), and uric acid transporter 1 (URAT1) expression (30). Moreover, Huining Dai et al. reported that sunflower head extract not only reduced UA levels in a manner comparable to established pharmacological agents such as allopurinol and benzbromarone but also diminished urea concentrations and facilitated the repair of uric acid-induced renal damage (23). Collectively, these studies advocate for the potential utility of sunflower seed head extract as an alternative therapeutic option for managing HUA.

The prevalence of HUA is on an upward trajectory within the Chinese population (31). Nonetheless, the clinical deployment of current first-line urate-lowering therapies is hampered by their narrow therapeutic indications and a spectrum of adverse effects, including gastrointestinal disturbances, cardiovascular complications, long-term hepatorenal toxicity, and the propensity to trigger acute gouty attacks (32). In light of these challenges, previous foundational research and clinical observations have identified the KYCT) treatment as efficacious in reducing uric acid levels, exerting anti-inflammatory and analgesic effects, and facilitating the restoration of hepatic and renal function. Importantly, KYCT has been characterized by an excellent safety profile, devoid of significant adverse reactions. Consequently, the outcomes of our investigation underscore the promising role of KYCT in the management of HUA and gout, advocating for its therapeutic consideration.

KYCT’s therapeutic potential is anchored in its dual capacity to achieve biochemical efficacy and clinically meaningful outcomes. The primary endpoint (sUA <6 mg/dL), aligned with ACR guidelines. Our anticipated 60-65% target attainment rate mirrors botanical precedents [58-72% (26)] and approaches first-line agents (29). Beyond urate lowering, KYCT’s multicomponent design may confer metabolic advantages, with preclinical data suggesting reductions in fasting glucose and LDL cholesterol, which is critical for hyperuricemia patients with metabolic comorbidities (33). Importantly, KYCT’s safety profile positions it as a viable alternative for high-risk populations. Future large-scale trials will utilize FDA-endorsed endpoints (e.g., tophus resolution, annualized flare rates) to definitively establish comparative effectiveness against standard therapies.

To translate KYCT’s therapeutic potential into clinically meaningful outcomes, we prespecified a minimal clinically important difference (MCID) threshold (≥1.2 mg/dL sUA reduction)—anchored to gout flare risk reduction (28) and renal protection benchmarks (29). This addresses a critical gap in botanical trials lacking pharmacodynamic standards. Demonstrating MCID attainment rates comparable to first-line agents [allopurinol: 1.5-2.0 mg/dL; febuxostat: 1.8-2.3 mg/dL (29) will inform future studies evaluating clinically anchored endpoints such as tophus volume reduction and CKD progression trajectories. Strategically, the threshold balances symptom mitigation (≥1.0 mg/dL) with a safety buffer (>3 mg/dL) to avoid overtreatment risks (5). A future biorepository substudy will correlate MCID achievement with multi-omics profiles to identify predictive biomarkers of therapeutic response.

In addition, compared to previous studies with smaller sample sizes from a single data source, this randomized controlled trial has several strengths, including its relatively expansive sample size and the employment of multicenter recruitment strategies, both of which contribute to the enhanced generalizability of the research findings. Furthermore, the methodological rigor is bolstered by the adoption of a double-blind, placebo-controlled design, intended to significantly reduce the potential for bias. The study’s design also includes evaluations at various intervals throughout a 12-week period to assess efficacy comprehensively, alongside the implementation of dietary controls to more real-world clinical practice.

Nonetheless, there are some limitations. The 12-week duration is relatively short, which precludes evaluation of long-term efficacy and safety. Concurrent lifestyle modifications in both groups may confound effects attributed solely to the KYCT. Moreover, the study is likely underpowered to detect rare adverse events. Nevertheless, this rigorous trial design will provide valuable evidence on the efficacy and safety of KYCT for lowering uric acid levels in patients with HUA in the short term. We assume that future studies with longer follow-ups in a more diverse population will build on these findings.
